# Supplementary material for: EOGT enables residual Notch signaling in mouse intestinal cells lacking POFUT1
Source: Sci Rep. 2023 Oct 14;13:17473. doi: 10.1038/s41598-023-44509-5 (PMC10576774; doi:10.1038/s41598-023-44509-5)
Supplement: Supplementary file 1 — Supplementary Information 1. [file 41598_2023_44509_MOESM1_ESM.pdf]

EOGT Enables Residual Notch Signaling in Mouse Intestinal Cells Lacking POFUT1

Mohd Nauman [1], Shweta Varshney [1,2] Jiahn Choi [1,3], Leonard H. Augenlicht [1,3] and Pamela Stanley [1],*

[1] Dept. Cell Biology, Albert Einstein College of Medicine, New York, NY 10461

[2] Current address: Dudnyk, 5 Walnut Grove Drive, Suite 300, Horsham, PA 19044

[3] Depts. Medicine and Oncology, Albert Einstein College of Medicine, New York, NY 10461

Supplementary Files – Table of Contents

1. Supplementary Tables

Supplementary Table S1

Supplementary Table S2

2. Supplementary Figures

Supplementary Figure S1

Supplementary Figure S2

Supplementary Figure S3

Supplementary Figure S4

Supplementary Figure S5

Supplementary Figure S6

Supplementary Figure S7

Supplementary Figure S8

Supplementary Figure S9

Supplementary Table S1. Primers used in this study.

Gene	Name	Sequence 5'-3'
**CHO**		
Eogt	For: 2F<br>Rev: 10R	*CCACTGACCTACATGCCCACCCTTAAA*<br>*CAACAGAAACCTATGAAGTTAGGGTTGTCTGG*
Eogt	For: SV20F<br>Rev: SV20R	*ATGTTAATGCTGCTTGCCTTTGGAG*<br>*CTGAAAGAATTGTTCACGTGCTGGG*
**Mouse**		
Pofut1 F/del	For: PS644<br>Rev: PS645	*GGGTCACCTTCATGTACAAGTGAGTG*<br>*ACCCACAGGCTGTGCAGTCTTTG*
Pofut1 F/WT	For: FB21<br>Rev: FB22	*CCAGGCTGATCACTTCTTGG*<br>*CCCTGTCTCGAAAAAGCAAA*
Eogt	For: 3rd loxF<br>Rev: 3rd loxR<br>Rev: 25307 Rv	*CCACCCGACCCCTGCCAGAACATAATGCTCTCTTGCATC*<br>*GCTGTCGCCAGAGGAGAGAGTGGGTGCTTACTTAC*<br>*CCAAGGCGGTCTTGGCCCAT*
Villin-Cre	For: 16775<br>Rev: 16776<br>Rev: oIMR9074	*GCCTTCTCCTCTAGGCTCGT*<br>*TATAGGGCAGAGCTGGAGGA*<br>*AGGCAAATTTTGGTGTACGG*
Hes1	For<br>Rev	*AGCTGGAGAGGCTGCCAAGGTTT*<br>*ACATGGAGTCCGAAGTGAGCGAG*
Hes5	For<br>Rev	*GGA CCA GAG GAT GAG CTC GTT*<br>*AGG AGG GAG CCT TCG GAA GA*
Hes7	For<br>Rev	*GAGCGAGCTGAGAATAGGGA*<br>*GGCTTCGCTCCCTCAAGTAG*
Hey1	For<br>Rev	*TGAGCTGAGA AGGCTGGTAC*<br>*ACCCCAAACTCCGATAGTC*
Math1	For<br>Rev	*ATGCACGGGCTGAACCA*<br>*TCGTTGTTGAAGGACGGGATA*
Notch2	For<br>Rev	*TGTACCAGATCCCAGAGATGC*<br>*GTCAGATGCAGAGTGTGGTGA*
Dll1	For<br>Rev	*GTCTGCCAGGGTGTGATGAC*<br>*CGGATGCACTCATCGCAGTA*

Dll4	For<br>Rev	*AGTGCCAGAACAGAGGTCCAA*<br>*CAGGGACTTCGGGCACAAT*
Jag1	For<br>Rev	*TGACATGGATAAACACCAGCA*<br>*GCAGCCCACTGTCTGCTATAC*
Jag2	For<br>Rev	*ATTGTAGCAAGGTATGGTGCG*<br>*GCACAGTTGTTGTCCAAATGA*
Lgr5	For<br>Rev	*TCTCCTACATCGCCTCTGCT*<br>*TTCCTCCGGAACCTGTCTCA*
Olfm4	For<br>Rev	*ATTCGCTATGGCCAAGGAGG*<br>*GAGGGGCCGATTCACATCAA*
Chga	For<br>Rev	*GAAGTGCG CCTGGAAGTCA*<br>*GATCCTCTCGTCTCCTTGGA*
Fabp2	For<br>Rev	*CTAGAGACACACACAGCTGAGATCATGG*<br>*GCAATCAGCTCCTTTCCATTGTCTACAC*
Hprt	For<br>Rev	*TCAGTCAACGGGGGACATAAA*<br>*GGGGCTGTACTGCTTAACCAG*
Gapdh	For<br>Rev	*GTGTCCGTCGTGGATCTGA*<br>*CCTGCTTCACCACCTTCTTG*

Supplementary Table S2. Antibodies used in this study.

Antibody	Fluorochrome	Isotype	Clone	Cat. no.	Company
NOTCH1 ECD	-	Ag-purified polyclonal sheep	aa19-526	AF5267	R&D Systems Inc., Minneapolis, MN
Cleaved NOTCH1	-	Rabbit IgG		4147	Cell Signaling Tech., Danvers, MA
Cleaved NOTCH2	-	Rabbit IgG	Polyclonal	93-7	G. A. Weinmaster, UCLA
CD45	PerCP	Rabbit IgG2b, κ	30-F11	103129	Biolegend, San Diego, CA
CD44	PE-Cyanine 7	Rabbit IgG2b, κ	IM7	25-0441-81	eBioscience, San Diego, CA
CD24	BV510	Rabbit IgG2b, κ	M1/69		Biolegend, San Diego, CA
CD166	Alexa Fluor 700	Goat IgG	Polyclonal	FAB1172N	R&D Systems Inc., Minneapolis, MN
GRP78	Alexa Fluor 647	Rabbit IgG	Polyclonal	PA1-014A-A647	Invitrogen, Carlsbad, CA
Anti-sheep IgG	Rhodamine Red-X	Donkey anti-sheep IgG	Polyclonal	713-295-147 or 713-295-003	Jackson ImmunoResearch, Lab. Inc., West Grove, PA
Anti-human Fcγ	Dylight™ 405	Goat anti-human IgG	Polyclonal	109-476-170	Jackson ImmunoResearch, Lab. Inc., West Grove, PA
Anti-human Fcγ	Alexa Fluor 488	Rabbit anti-human IgG	Polyclonal	A-21220	Molecular Probes, Eugene, OR
Anti-bovine POFUT1	-	Rabbit anti-bovine POFUT1	Polyclonal	Loriol et al. (2006) Glycobiology 16: 736-747	Not applicable

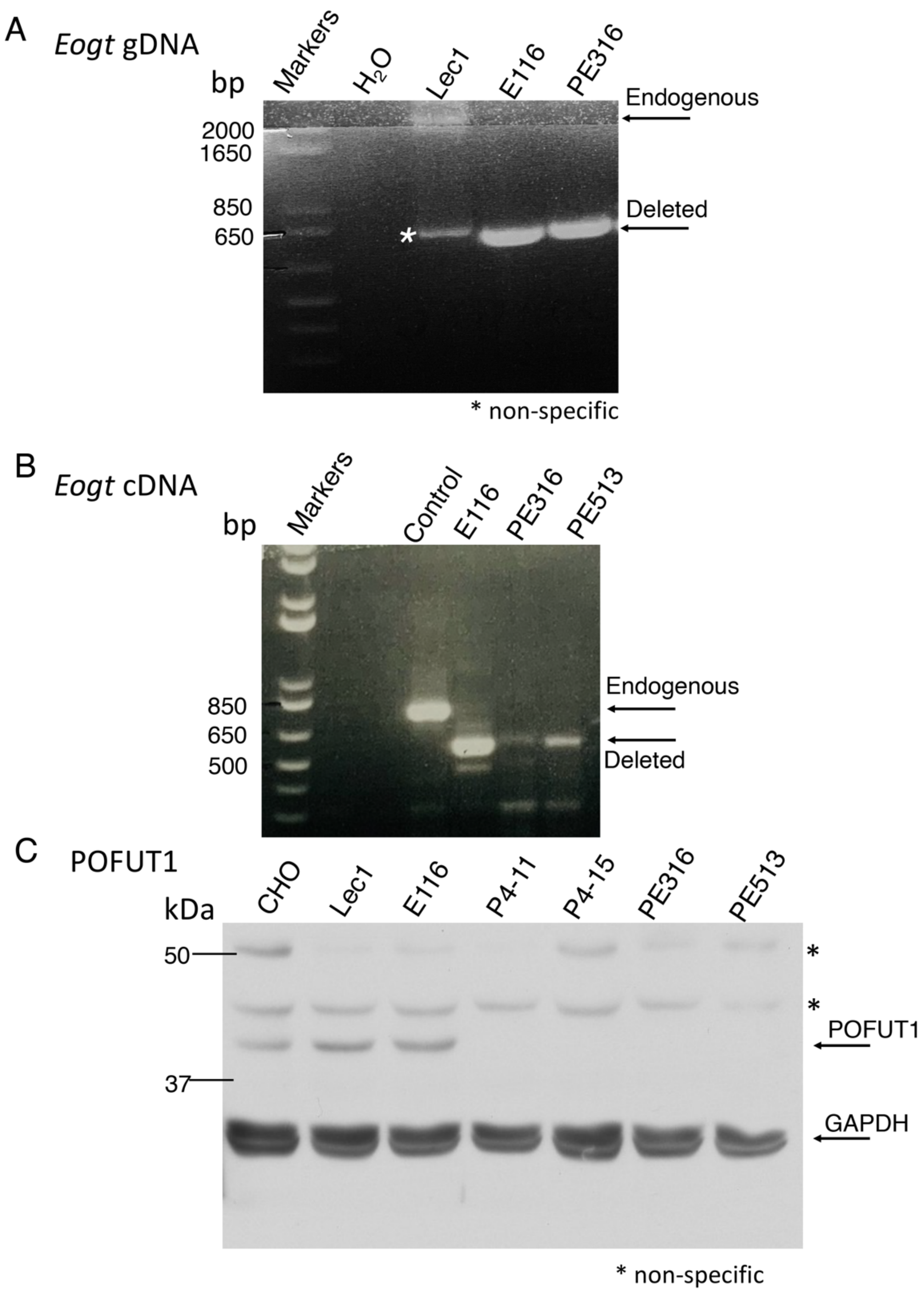

Supplementary Figure S1. Mutation of *Eogt* and *Pofut1* in Lec1 CHO cells. *(A)* PCR of genomic DNA (gDNA) to detect *Eogt* in E116 and PE316 cells. Endogenous *Eogt* in Lec1 cells (2198 bp) and the deleted band in E116 and PE316 cells (818 bp) are indicated. *(B)* RT-PCR of cDNA to detect endogenous *Eogt* cDNA (800 bp) in Lec1 cells and deleted band (614 bp) in E116 and PE316 cells. *(C)* Western blot analysis showing POFUT1 expression in CHO, Lec1 and E116 cells and no band for POFUT1 in P4-11, P4-15, PE316 and PE513 cells.

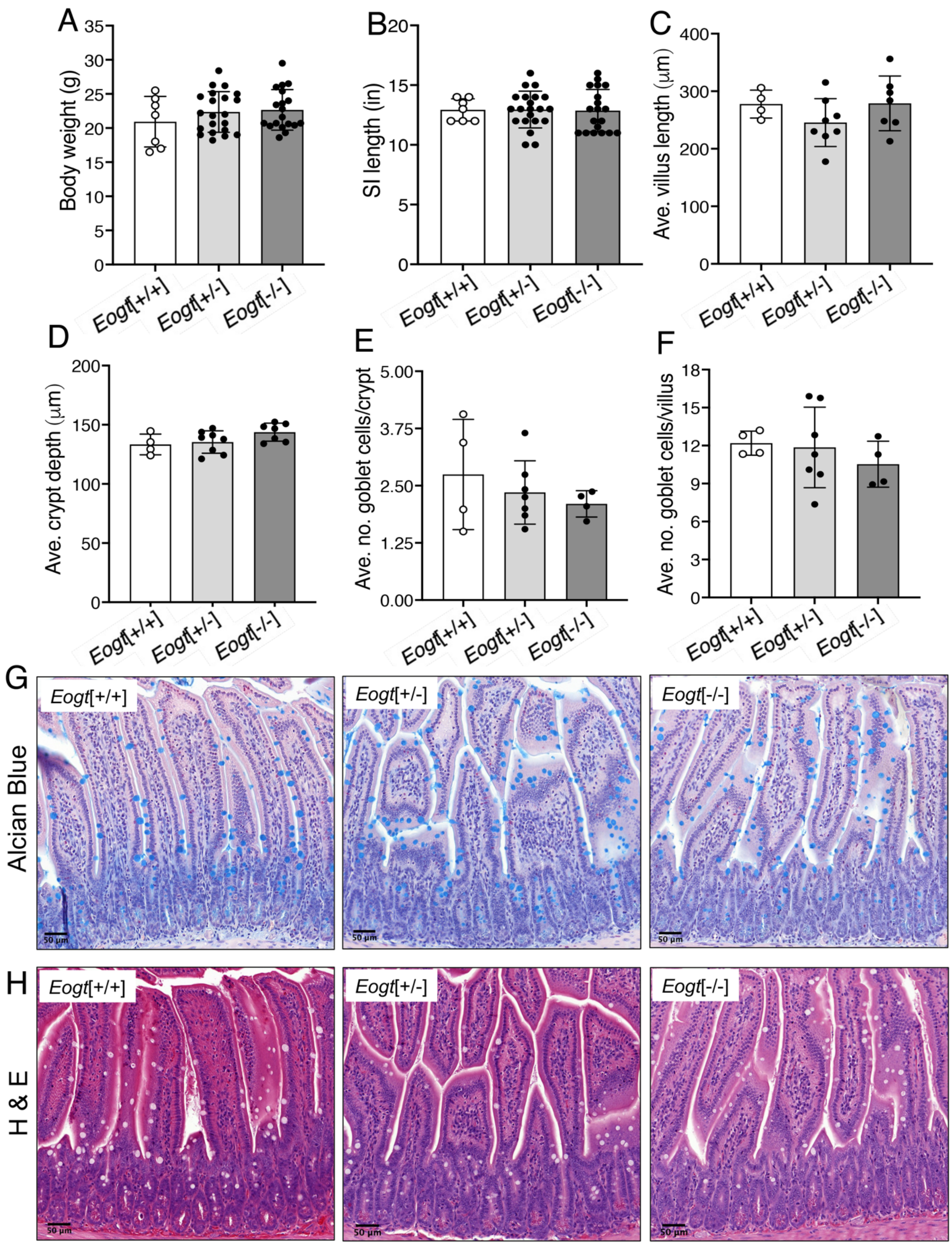
A
Body weight (g)
B
SI length (in)
C
Ave. villus length (μm)
D
Ave. crypt depth (μm)
E
Ave. no. goblet cells/crypt
F
Ave. no. goblet cells/villus
Eogt[+/+]
Eogt[+/-]
Eogt[-/-]
G
Alcian Blue
H
H & E

Supplementary Figure S2. *Eogt* deletion has no apparent effects on overall differentiation in the intestinal epithelium. (*A-F*) Comparison between *Eogt*[+/+], *Eogt*[+/-] and *Eogt*[-/-] of body weight (n $\geq$ 7 mice per group), length of small intestine (n $\geq$ 7 mice per group), villi length (20 villi were analyzed in 4 mice per group), crypt depth (20 crypts were analyzed in 4 mice per group) and, number of goblet cells in villi and crypts (30 villi and 100 crypts were analyzed in 4 mice per group). (*G* and *H*). Representative images (n = 4 mice per group) showing goblet and Paneth cells in small intestine. Scale bar: 50 μm.

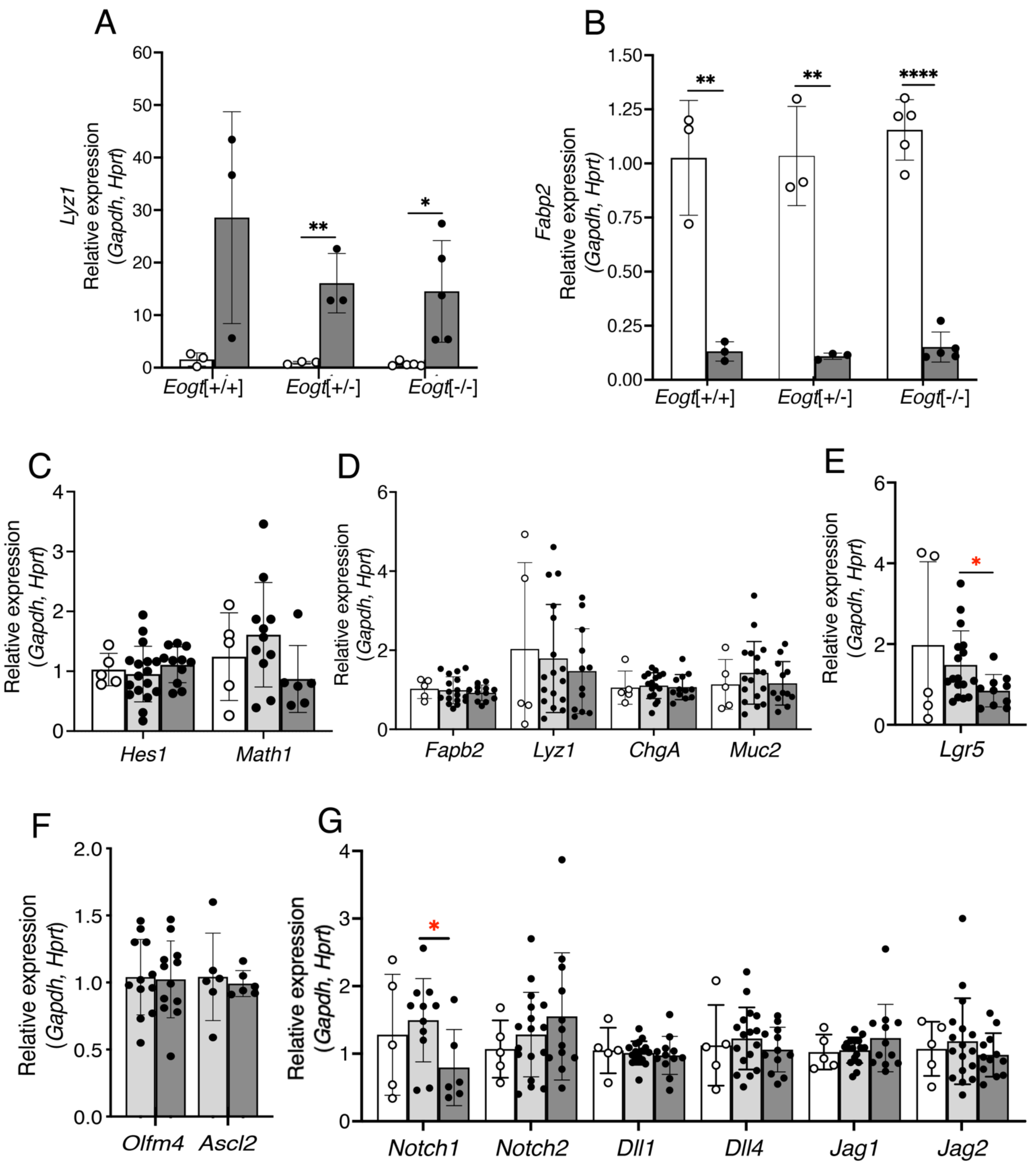
A
Lyz1
Relative expression
(Gapdh, Hprt)
0
10
20
30
40
50
60
**
*
Eogt[+/+]
Eogt[+/-]
Eogt[-/-]
B
Fabp2
Relative expression
(Gapdh, Hprt)
0.00
0.25
0.50
0.75
1.00
1.25
1.50
**
**
****
Eogt[+/+]
Eogt[+/-]
Eogt[-/-]
C
Relative expression
(Gapdh, Hprt)
0
1
2
3
4
Hes1
Math1
D
Relative expression
(Gapdh, Hprt)
0
2
4
6
Fapb2
Lyz1
ChgA
Muc2
E
Relative expression
(Gapdh, Hprt)
0
2
4
6
*
Lgr5
F
Relative expression
(Gapdh, Hprt)
0.0
0.5
1.0
1.5
2.0
Olfm4
Ascl2
G
Relative expression
(Gapdh, Hprt)
0
1
2
3
4
*
Notch1
Notch2
Dll1
Dll4
Jag1
Jag2

Supplementary Figure S3. Validation of villi and crypts fractionation and expression of Notch target and pathway genes in *Eogt* mutant mice. (*A* and *B*) Enrichment of *Lyz1* in crypts (gray) and *Fabp2* in villi (white) is shown for *Eogt*[+/+], *Eogt*[+/-] and *Eogt*[-/-] (n ≥ 3 mice per group). (*C-G*) Quantitative RT-PCR analysis of Notch signaling related genes in the small intestine in mice with partial or full deletion of *Eogt*. Transcript levels of Notch targets, markers for absorptive and secretory lineages, ISC marker(s), Notch receptors and Notch ligands in *Eogt*[+/+] (white), *Eogt*[+/-] (light gray) and *Eogt*[-/-] (light gray) (n ≥ 5 mice per group). P values were determined by one-way ANOVA with Tukey's correction *P < 0.05, **P < 0.01, ****P < 0.0001 or unpaired, two-tailed Student's t test with Welch's correction - *P < 0.05.

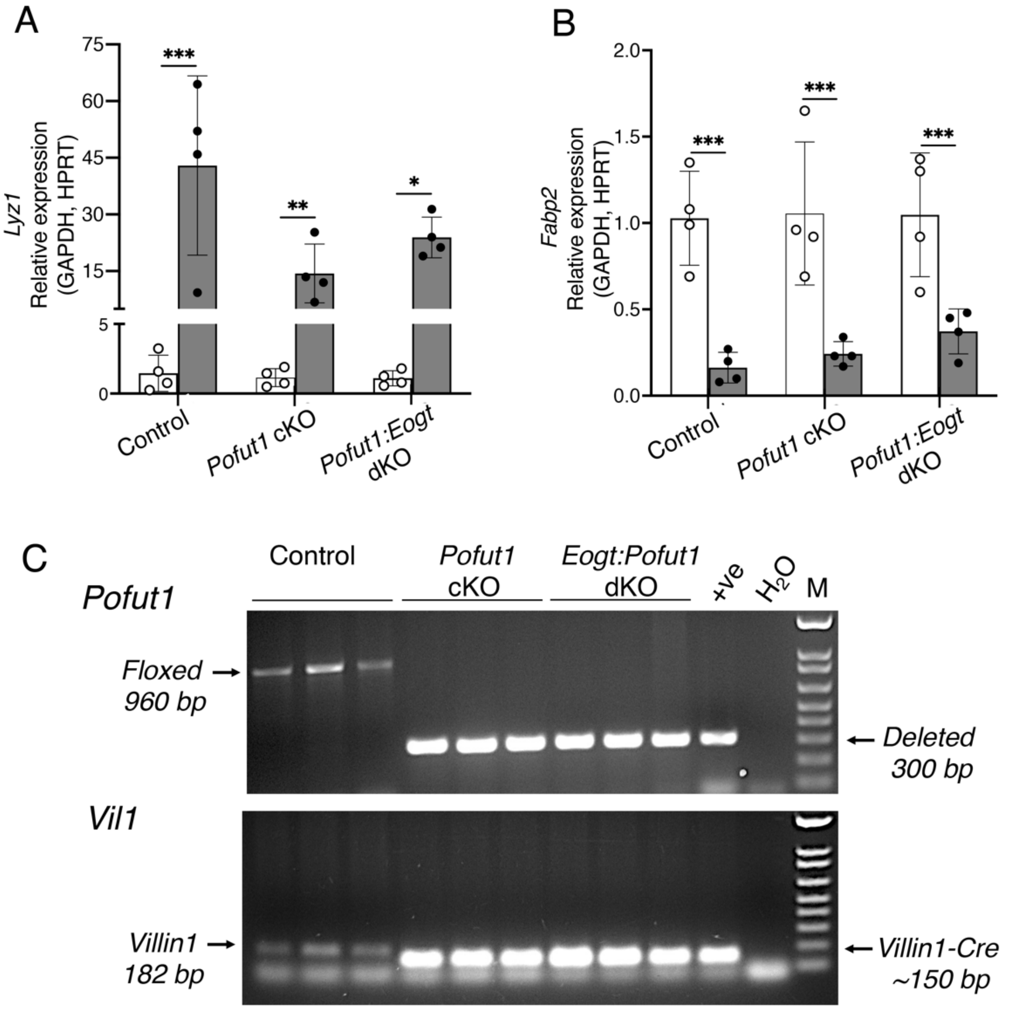

Supplementary Figure S4. Validation of isolated villi and crypts fractionation scheme. (*A* and *B*) Enrichment of *Lyz1* in crypts (gray) and *Fabp2* in villi (white) are shown in Control, *Pofut1* cKO and *Eogt:Pofut1* dKO fractions (n ≥ 4 mice per group). P values were determined by one-way ANOVA with Tukey's correction - *P < 0.05, **P < 0.01, ***P < 0.001 ****P < 0.0001. (*C*) Villin1-Cre deletion of *Pofut1* (300 bp) is shown in gDNA isolated from crypts of *Pofut1* cKO and *Eogt:Pofut1* dKO mice, and *Pofut1* floxed band (960 bp) is shown in gDNA from crypts of control mice (n = 3 mice per group). Controls were a known positive (+ve) and H_2O. M denotes 100 bp markers. The Villin1 endogenous gene (182 bp) and Villin-Cre transgene (~150 bp) were detected in Control versus *Pofut1* cKO and *Eogt:Pofut1* dKO mice (n = 3 mice per group). The endogenous gene was difficult to detect in heterozygous transgenic mice due to the robust production of the smaller PCR product from the transgene.

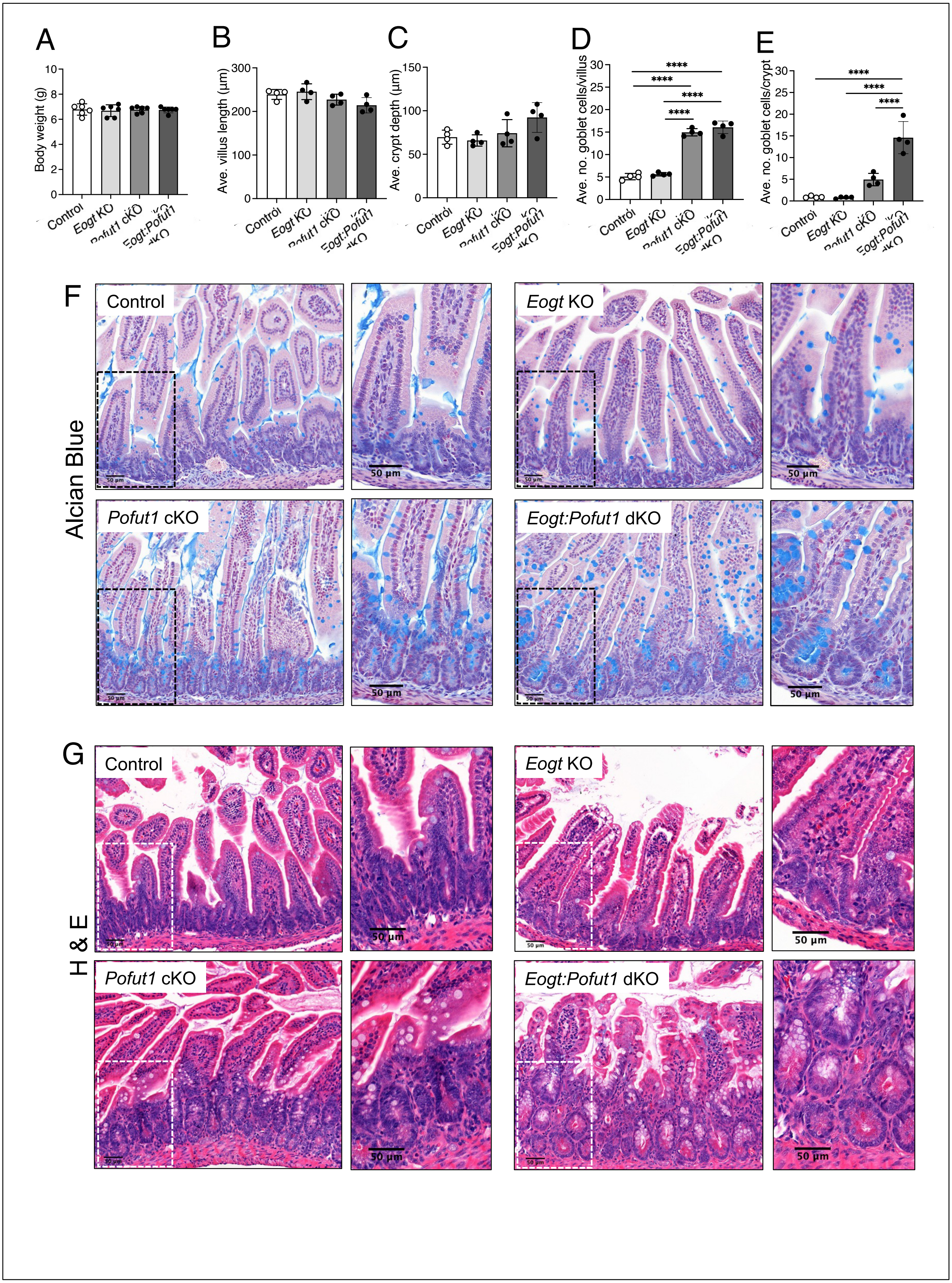
A
B
C
D
E
Body weight (g)
Ave. villus length (µm)
Ave. crypt depth (µm)
Ave. no. goblet cells/villus
Ave. no. goblet cells/crypt
Control
Eogt KO
Pofut1 cKO
Eogt:Pofut1 dKO
****
F
Alcian Blue
G
H & E
50 µm

Supplementary Figure S5. Effects of combined deletion of *Pofut1* and *Eogt* on small intestine at P15. (*A*-*C*) Body weight (n = 6 mice per group), villus length and crypt depth of Control, *Eogt* KO, *Pofut1* cKO and *Eogt:Pofut1* dKO (20 villi or 20 crypts were analyzed in 4 mice per group). (*D* and *E*) Number of goblet cells in villi and crypts of experimental groups (30 villi and 100 crypts were analyzed in 4 mice per group). (*F* and *G*) Representative images showing goblet and Paneth cells in the small intestine of experimental mice (n = 4 mice per group). Boxed areas show enlarged images to the right of each panel. P values were determined by one-way ANOVA with Tukey's correction - ****P < 0.0001. Scale bar : 50 μm.

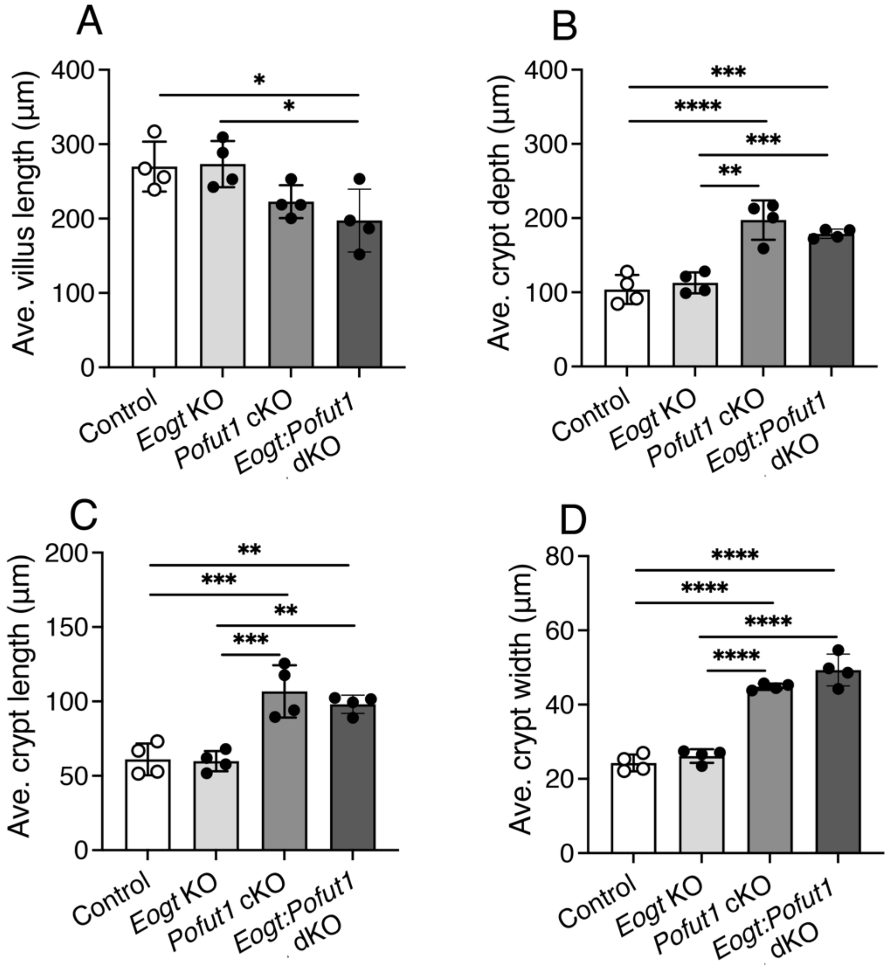

Supplementary Figure S6. Effects of *Pofut1* deletion with and without *Eogt* at P28. (*A*-*D*) Comparisons of villi length, crypt depth, crypt length and crypt width in Control, *Eogt* KO, *Pofut1* cKO and *Eogt:Pofut1* dKO (n = 4 mice per group). P values were determined by one-way ANOVA with Tukey's correction - *P < 0.05, **P < 0.01, ***P < 0.001, ****P < 0.0001.

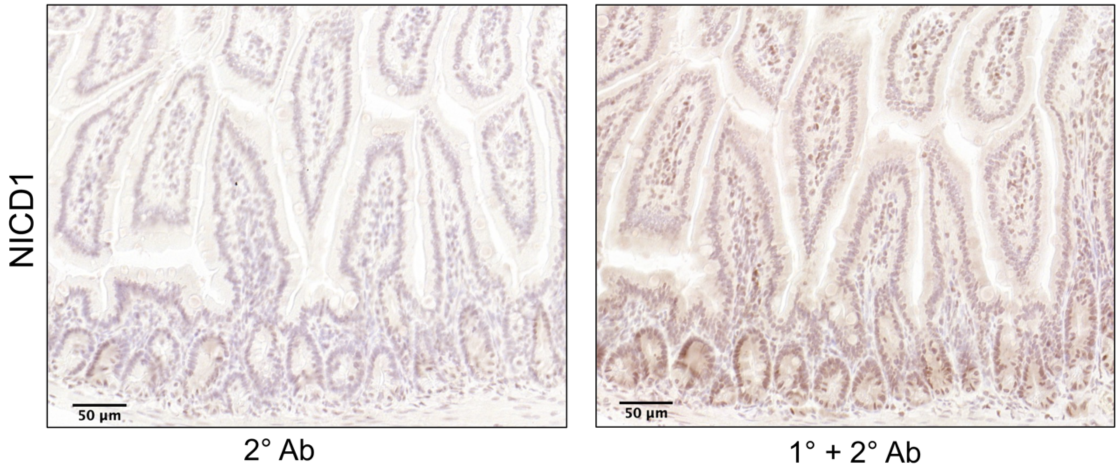

Supplementary Figure S7. Controls for western blotting analysis. (*A* and *B*) Small intestine tissues from Control mice showing signals with and without primary antibodies for NICD1 by IHC. Scale bar : 50 μm.

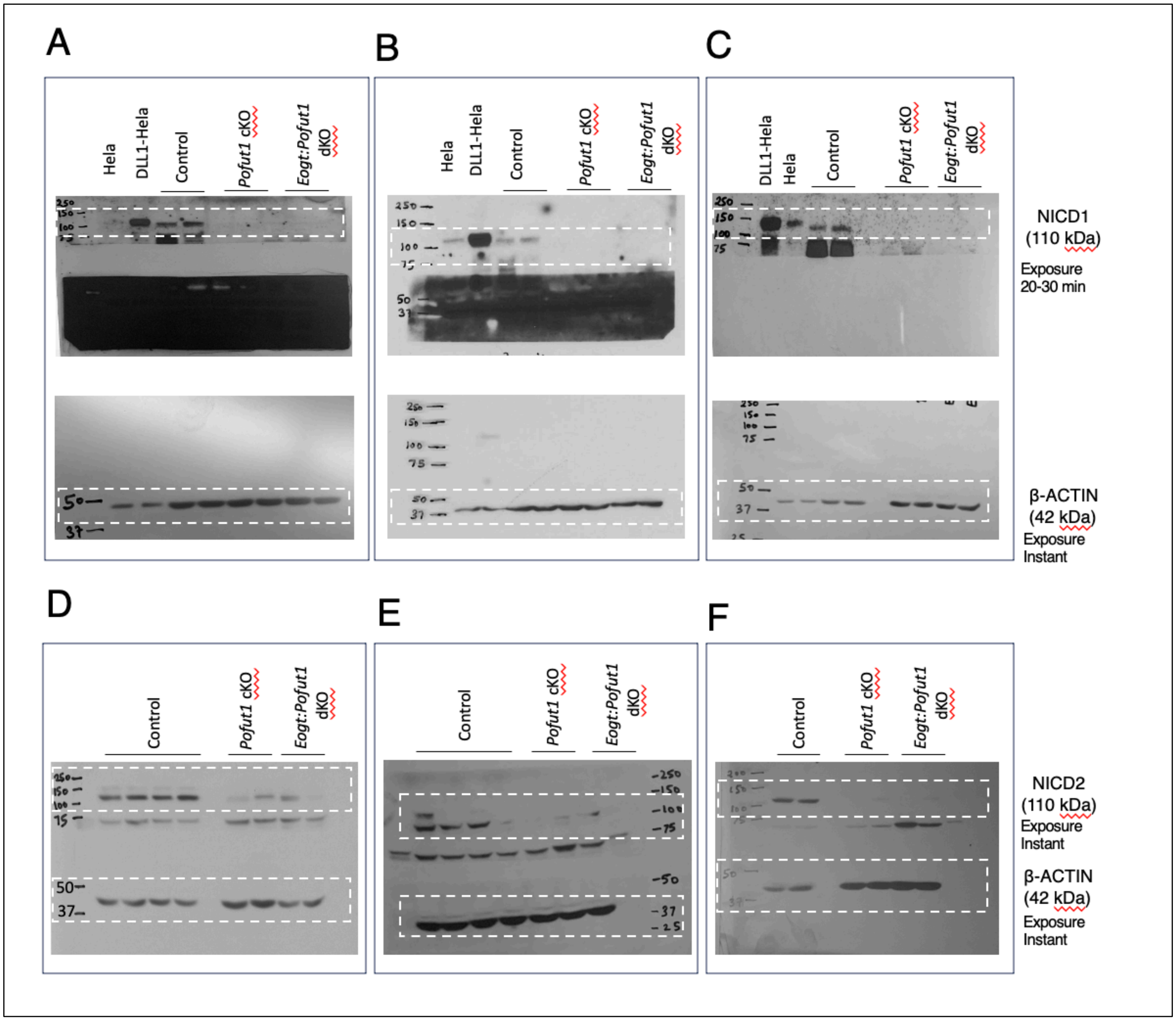

Supplementary Figure S8. Expression of activated NOTCH receptors in small intestine. (*A-C* and *D-F*). Full length Western blots of replicates performed for NICD1 and NICD2 in crypts of Control, *Pofut1* cKO and *Eogt:Pofut1* dKO intestine. Portions of Fig. S8A and S8D are shown in Fig. 4B. Following transfer of gels to PVDF membrane, blots were blocked in Tris-buffered non-fat dry milk and then cut at the mid-point between the 75 and 50 kDa visible molecular weight markers. Each section was incubated separately with relevant antibodies. The membranes were placed with a gap between them in an X-ray cassette. The NICD1 blots were exposed to film for 20 or 30 min. Subsequently, membranes were exposed for an instant for the accompanying β-actin blot. The NICD1 gel contained controls for endogenous NICD1 (low level in HeLa extract) and extract from DLL1-induced HeLa cells with an increased level of NICD1. These controls were cropped from Fig. 4B. The NICD2 blot and its accompanying β-actin blot were both exposed for an instant. Dotted lines highlight specific signals.

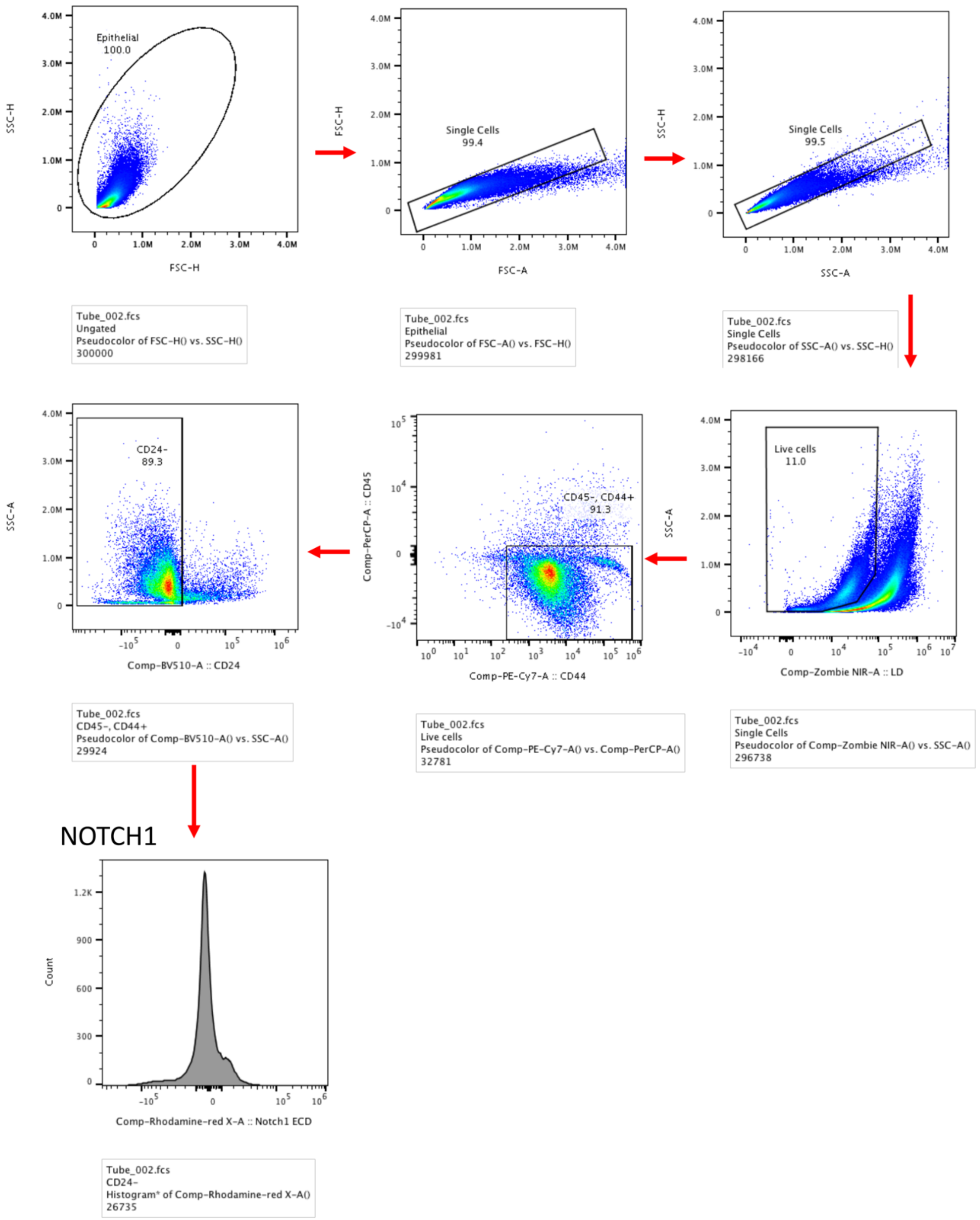

Supplementary Figure S9. Control mouse showing gating strategy to analyse binding of anti-NOTCH1 ECD or different soluble Notch ligands in ISC of experimental mice. Single live cells were gated to select CD45$^-$CD44$^+$CD24$^-$ cell population. CD45$^-$CD44$^+$CD24$^-$ cells were used to calculate Mean fluorescence index (MFI) for anti-NOTCH1 binding or only 2° Ab sample. Similar gating strategy was used to determine DLL1-Fc or DLL4-Fc binding to ISCs.
