# Supplementary material for: EOGT enables residual Notch signaling in mouse intestinal cells lacking POFUT1
Source: Sci Rep. 2023 Oct 14;13:17473. doi: 10.1038/s41598-023-44509-5 (PMC10576774; doi:10.1038/s41598-023-44509-5)
Supplement: Supplementary file 2 — Supplementary Information 2. [file 41598_2023_44509_MOESM2_ESM.pdf]

EOGT Enables Residual Notch Signaling in Mouse Intestinal Cells Lacking POFUT1

Mohd Nauman [1], Shweta Varshney [1,2] Jiahn Choi [1,3], Leonard H. Augenlicht [1,3] and Pamela Stanley [1]

[1] Dept. Cell Biology, Albert Einstein College of Medicine, New York, NY 10461
[2] Current address: Dudnyk, 5 Walnut Grove Drive, Suite 300, Horsham, PA 19044
[3] Depts. Medicine and Oncology, Albert Einstein College of Medicine, New York, NY 10461

Supplemental Information on western blots in Figure 4C.

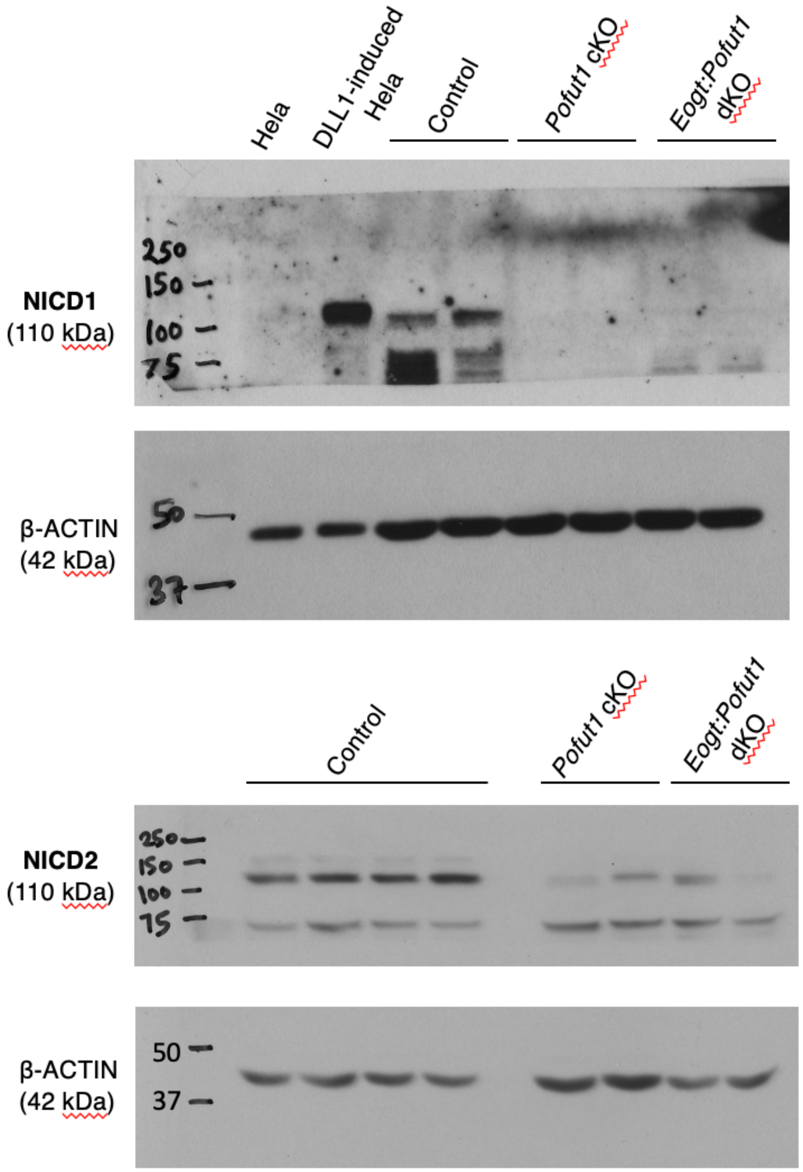

Following transfer of gels to PVDF membrane, blots were blocked in Tris-buffered non-fat dry milk and then cut at the mid-point between the 75 and 50 kDa visible molecular weight markers. Each section was incubated separately with relevant antibodies. The NICD1 blot was exposed to film for 20 min, the accompanying β-actin blot was exposed for an instant. The NICD2 blot and its accompanying β-actin blot were both exposed for an instant.

The NICD1 gel contained controls for endogenous NICD1 (low level in HeLa extract) and extract from DLL1-induced HeLa cells with a high level of NICD1. These controls were cropped in Fig. 4C.
